# Dyslipidemia initiates keratinocytes proliferation through upregulation of lncRNA NEAT in psoriasis patients

**DOI:** 10.1007/s11033-023-08527-w

**Published:** 2023-08-02

**Authors:** Abeer Mostafa, Dina Sabry, Nesreen Aboraia, Ahmed Fawzy, Amany A. Abou-Elalla

**Affiliations:** 1 Medical Biochemistry and Molecular Biology department, Faculty of Medicine, Cairo University, Cairo, Egypt; 2 Medical Biochemistry and Molecular Biology department, Faculty of Medicine, Badr University in Cairo, Badr City, Egypt; 3 Dermatology department, Faculty of Medicine, Fayoum University, Fayoum, Egypt; 4 Physiology department, Faculty of Medicine, Cairo University, Cairo, Egypt; 5 Medical Laboratory Technology department, Faculty of Applied Health Science Technology, Misr University for Science and Technology, 6th of October City, Egypt

**Keywords:** Psoriasis, NEAT, TNF-α, VEGF, ROS, Caspase-3

## Abstract

**Background:**

Psoriasis is a chronic inflammatory immune-mediated and hyper proliferative skin disorder that has underlying genetic factors. Psoriasis can result from interaction of cytokines between keratinocytes and T-lymphocytes. NEAT is a lncRNA involved in immune modulation and has been previously studied in cancers. This study aims to clarify the unprecedented role of NEAT in psoriasis pathogenesis.

**Methods:**

The study was conducted on 50 healthy control subjects and 50 psoriasis patients. Blood samples from all participants were collected for analysis of their lipid profile. qRT-PCR was done for lncRNA NEAT, TNF-α, VEGF genes expression. The levels of ROS and caspase-3 were estimated by ELISA. ROC analysis was done to detect the diagnostic value of lncRNA NEAT gene expression.

**Results:**

Dyslipidemia is more prevalent among psoriasis patients. A significant up regulation in lncRNA NEAT, TNF-α, VEGF genes expression (*p* value˂0.001) in psoriasis patients in addition to significant increase in ROS and caspase-3 levels (*p* value˂0.001) in compare to controls. Additionally, a positive significant correlation between TNF-α, ROS, NEAT, caspase-3 and dyslipidemia. NEAT had an area under the curve (AUC) of 0.931 (95% CI 0.844–0.978, *p* < 0.001).

**Conclusion:**

Dyslipidemia is an initiating signal in psoriasis pathogenesis that creates a state of chronic inflammation and oxidative stress. This state induces keratinocytes proliferation and release of NEAT with subsequent caspase-3 activation to counteract the proliferating cells. NEAT could be considered as a good diagnostic biomarker for psoriasis.

**Graphical abstract:**

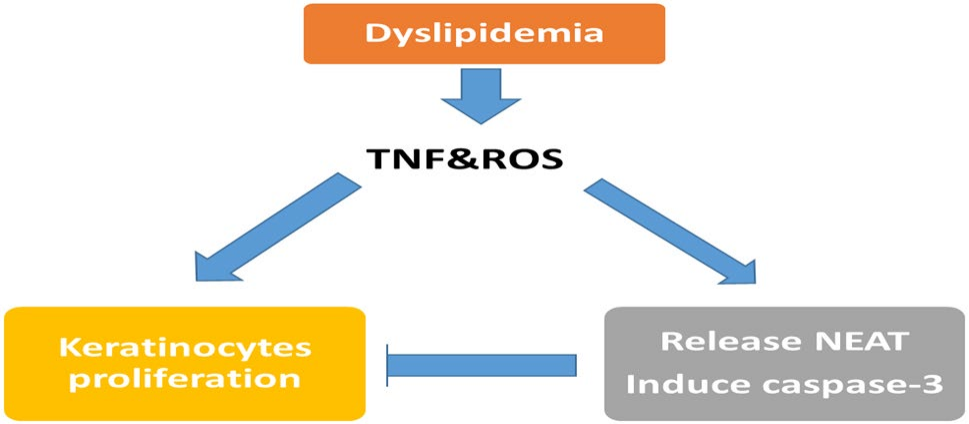

## Introduction

 Psoriasis is a chronic inflammatory disease affects mainly joint and skin. Psoriasis has bad emotional and psychosocial impact on patients [1]. Psoriasis is characterized by excessive proliferation of keratinocytes and infiltration of the immune cells in the epidermis and dermis [2].

In the past several years; many researches were developed on long non-coding RNAs (lncRNAs) due to its vital role in biological process regulation [3]. LncRNAs have a crucial role in epigenetic regulation, by formation of networks composed of chromatin regulators and ribonucleoproteins [4].

Several previous studies revealed that lncRNAs are expressed in different stages of immune development, activation. LncRNAs had been shown to be involved in many diseases, such as autoimmune disorders including rheumatoid arthritis, systemic lupus erythematosus, and psoriasis. Recently it has been demonstrated that lncRNA NEAT acts as a novel regulatory factor in the immune system [5].

Paraspeckles are a huge network of protein-protein interaction that seemed to be hold together by the lncRNA NEAT with other cellular regulatory proteins [6].

Tumor necrosis factor-α (TNF-α) is a multifunctional cytokine. TNF plays an crucial role in immunological and inflammatory responses in human skin. It had been reported that TNF plays a vital role in psoriasis development. TNF-α could induce reactive oxgyen species (ROS) generation in human keratinocytes, IκB degradation with subsequent nuclear translocation of nuclear factor kappa B (NF-κB), p65, and inflammatory cytokines production [7].

Oxidative stress is only partly responsible for psoriasis, however in general it can be stated that excessive oxidative is present in patients suffering from psoriasis. ROS are generated from both external pro-oxidant stimuli and endogenous neutrophils, these results in increased ability of chemotaxis, adhesion, and fibroblasts activation. This is approved high MDA plasma levels and decreased the antioxidant defense enzymes levels in psoriasis patients [8].

Active caspase enzyme can mediate pro-inflammatory cytokines proteolytic maturation and induce apoptosis. Caspases activation is crucial for the defense against damaged cells and pathogens, their dysregulation could contribute to cancer, autoimmune, and neurodegenerative diseases [9].

NEAT1 stabilizes the mature caspases to promote cytokines production and initiates apoptosis. Inflammatory signals induce NEAT1 activation through its dissociation and translocation from the paraspeckles to the cytoplasm to affect the inflammatory and immune cells [10].

## Subjects and methods

### Study subjects

One hundred participants matched sex and age (ranging from 35 to 55) years were included in the study, according to ethical approval N-94-2023 obtained from Research Ethics Committee, Faculty of Medicine, Cairo University. An informed consent was obtained from all participants. The psoriasis patients were enrolled from the dermatology outpatient clinic, Faculty of Medicine, Cairo University. Sample size was calculated using the (G power software). Based on (Young et al.) [7] we found that 50 participants in each group was appropriate sample size with total sample size 100 participants (2 groups) The power is 80% and α error probability = 0.05, effect size f = 0.57.

The participants were divided into two: Group 1: 50 healthy controls. Group 2: 50 psoriasis patients. Five ml whole blood were withdrawn from all participants. Three ml were proceeded for RNA extraction followed by real time PCR for quantitative assessment of LncRNA NEAT, TNF-α and VEGF genes expression. The last two ml were centrifuged at 3000×*g* for 15 min to separate clear plasma and stored at − 80 °C for further assessment of ROS and Caspase-3 levels by ELISA. Additionally, Lipid profile was assessed in plasma using spectrophotometer. TAG (mg/dL), cholesterol (mg/dL), and HDL (mg/Dl) were measured.

### RNA extraction

From whole blood samples after about 2 weeks of samples storage in − 80°; total RNA was extracted from the two studied groups using Direct-zol ^TM^ RNA MiniPrep Catalog. No.: R2050. ZYMO RESEARCH according to the instructions of manufacturer. RNA purity abd quantitation assessment were carried out using spectrophotometer the Nano Drop® (ND)-1000 (Nano Drop Technologies, Inc. Wilmington, USA). For Quantitative Real-Time Polymerase Chain Reaction (qPCR);

One-Step Kit SensiFAST™ SYBR® Hi-ROX, Catalog No. PI-50217 V was used for cDNA synthesis and qPCR using a single tube in the 48-well plate Step One instrument (Applied Biosystem, USA) on a Rotor Gene Real-Time PCR System (Qiagen). Genes expressions were normalized to CAPDH by the ΔΔCt method. The thermal profile of the PCR run was adjusted as follows: 45 °C for 15 min in one cycle (for cDNA synthesis), 10 min at 95 °C for reverse transcriptase enzyme inactivation, followed by 40 cycles of PCR amplification. Each cycle in the form of 10 s at 95 °C, 30 s at 60 °C, and 30 s at 72 °C. the sequence of studied genes primers is listed in Table 1.

**Table 1:** primers sequence of all the studied genes

	Forward	Reverse
VEGF	5′-GAGATGAGCT TCCTACAGCAC-3′	5′-TCACCGCCTCGGCTTGTCACAT-3′
NEAT	5′-GTGGCTGTTGGAGTCGGTAT-3	5′-TAACAAACCACGGTCCATGA‐3′
TNF	5′- CTCTTCTGCCTGCTGCACTTTG-3	5′- ATGGGCTACAGGCTTGTCACTC-3
GAPDH	5′- GTCTCCTCTGACTTCAACAGCG-3	5′- ACCACCCTGTTGCTGTAGCCAA-3

### ELISA

The plasma levels of ROS (µg/ml) from the two studied groups were estimated using human ROS ELISA Kit. Catalog No: MBS251578. BioSource according to kits instructions.

The plasma levels of active caspase-3(µg/ml) from the two studied groups were estimated using human caspase 3 ELISA Kit, Catalog No: KHO1091. Labome according to kit instructions.

### Biochemical assessments

Measurements of lipid profile were carried out by colorimetric technique using reagent kits (total cholesterol Catalog No: CH 12 20, HDL Catalog No: CH 12 30, and triglyceride Catalog No: CH 20 30) Biodiagnostic Egypt.

### Statistical method

SPSS program version 22 was used for data analysis. Normality tests Kolmogorov-Smirnov and Shapiro-Wilk were used for exploration data normality and distribution. Data are presented as mean and standard deviation. Independent sample t test was used for comparison between the two studied groups regarding the numerical variables. χ^2^ test was used for qualitative data comparisons. Pearson correlation was used for the correlation between quantitative variable. Receiver operating characteristic (ROC) curve analysis was done to detect best diagnostic chosen cutoff value of LncRNA NEAT. P ≤ 0.05 is considered to be significant.

## Results

### Demographic and laboratory characteristics of psoriasis patients

The demographic and laboratory data analysis of the two studied groups revealed that there was no significant difference regarding the age and sex between the two studied groups (*p* value > 0.05). A significant increase in plasma TG and cholesterol and decrease in HDL levels in psoriasis patients when compared to the control subjects (*p* value = 0.008, < 0.001, < 0.001) respectively. Table 2.

**Table 2:** Demographic and biochemical laboratory data among the studied groups

Variable	Control Group (I) n = 50	Psoriasis Group (II) n = 50	*p* value
Sex			
Females: n (%) Males: n (%)	42 (42%) 58 (58%)	46 (46%) 54 (54%)	0.87
Age (years)	43.1 ± 10.9	45.7 ± 15.6	0.36
TG (mg/dl)	100.6 ± 26.2	127.3 ± 64.5*	0.008
Cholesterol(mg/dl)	116.7 ± 34.2	180.2 ± 45.7*	< 0.001
HDL	51.2 ± 8.5	35.8 ± 10.4*	< 0.001

Data were expressed as Mean ± SD, *p* value < 0.05 was significant

*Denotes significant difference versus control subjects

*TG* tri glyceride, *HDL* high density lipoprotein

### qRT PCR for lncRNA NEAT, VEGF and TNF-α genes expression

**Fig. 1:**
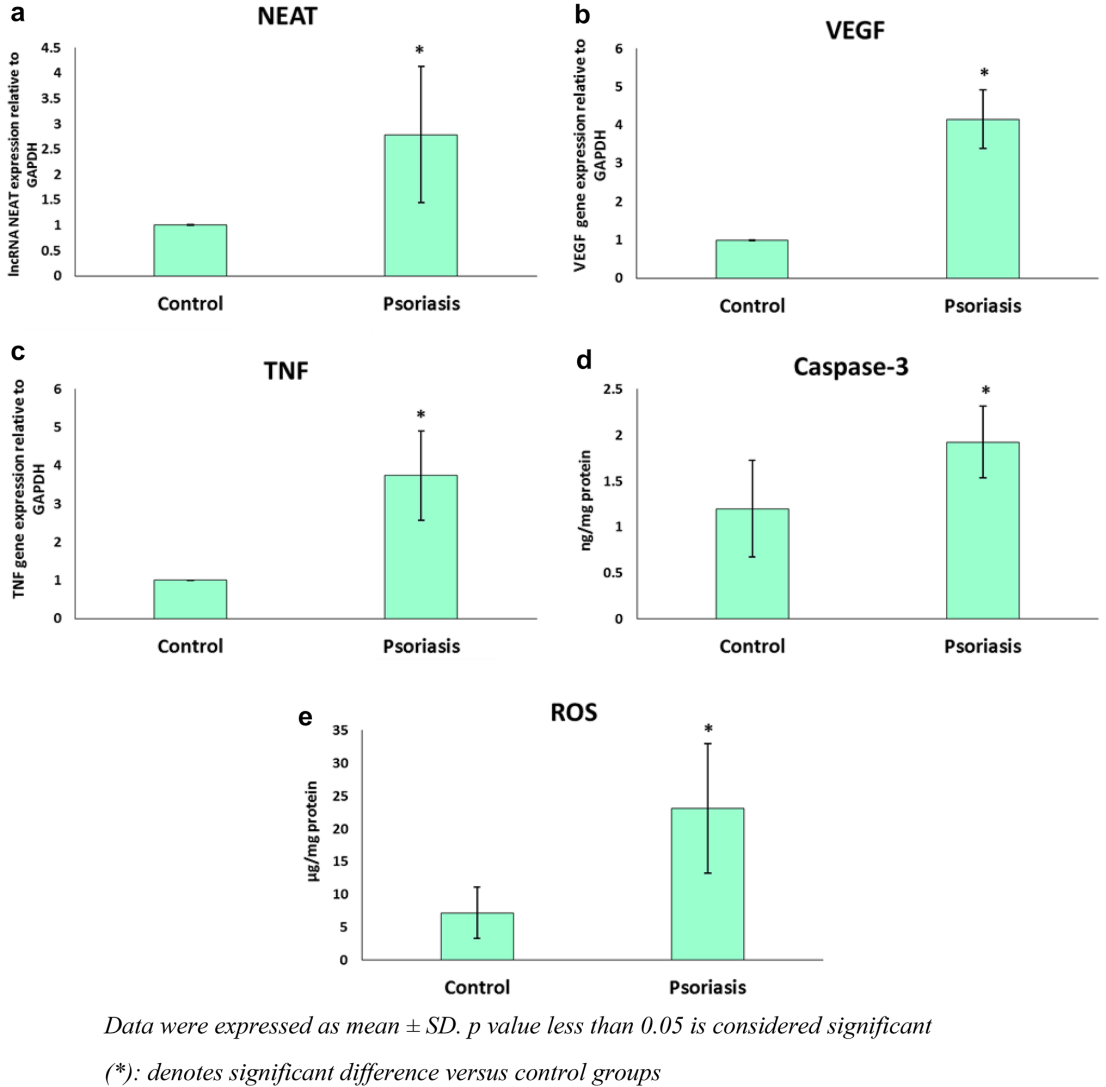
A statistically significant increase in all studied parameters (a-NEAT, b-VEGF, c-TNF-α, d-caspase 3 and e-ROS) in psoriasis patients as compared to controls. Data were expressed as mean ± SD. *p* value less than 0.05 is considered significant *denotes significant difference versus control groups(kindly insert the legend below the figure)

Regarding PCR results; a significant increase in lncRNA NEAT, VEGF and TNF-α genes expression in psoriasis patients as compared to healthy control subjects (*p* value < 0.001). Figure 1a–c respectively.

### ELISA for caspase-3 and ROS plasma levels

Regarding ELISA results; a significant increase in caspase-3 and ROS plasma levels in psoriasis patients while compared to healthy control subjects (*p* value < 0.001) Fig. 1d, e respectively.

### Correlation analysis between NEAT and other studied parameters with lipidemic state of psoriasis patients

Plasma cholesterol was significantly positively correlated with all studied parameters while HDL was inversely correlated with them (*p* value < 0.001). A significant positive correlation between ROS, TNF-α levels and NEAT and caspase-3 (*p* value < 0.001) as showed in Table 3.

**Table 3:** Correlation between all studied parameters

		TNF-α	NEAT	ROS	Caspase 3
TG	r	0.200*	0.028	0.096	0.128
	P value	0.046	0.780	0.341	0.204
Cholesterol	r	0.345**	0.301**	0.531**	0.471**
	P value	< 0.001	0.002	< 0.001	< 0.001
HDL	r	− 0.463**	− 0.519**	− 0.442**	− 0.340**
	P value	< 0.001	< 0.001	< 0.001	0.001
TNF-α	r	1	0.590**	0.463**	0.376**
	P value		< 0.001	< 0.001	< 0.001
NEAT	r	0.590**	1	0.473**	0.435**
	P value	< 0.001		< 0.001	< 0.001

* denotes that p value <0.05

** denotes that p value <0.01

### ROC analysis to determine the diagnostic performance of NEAT

**Fig. 2:**
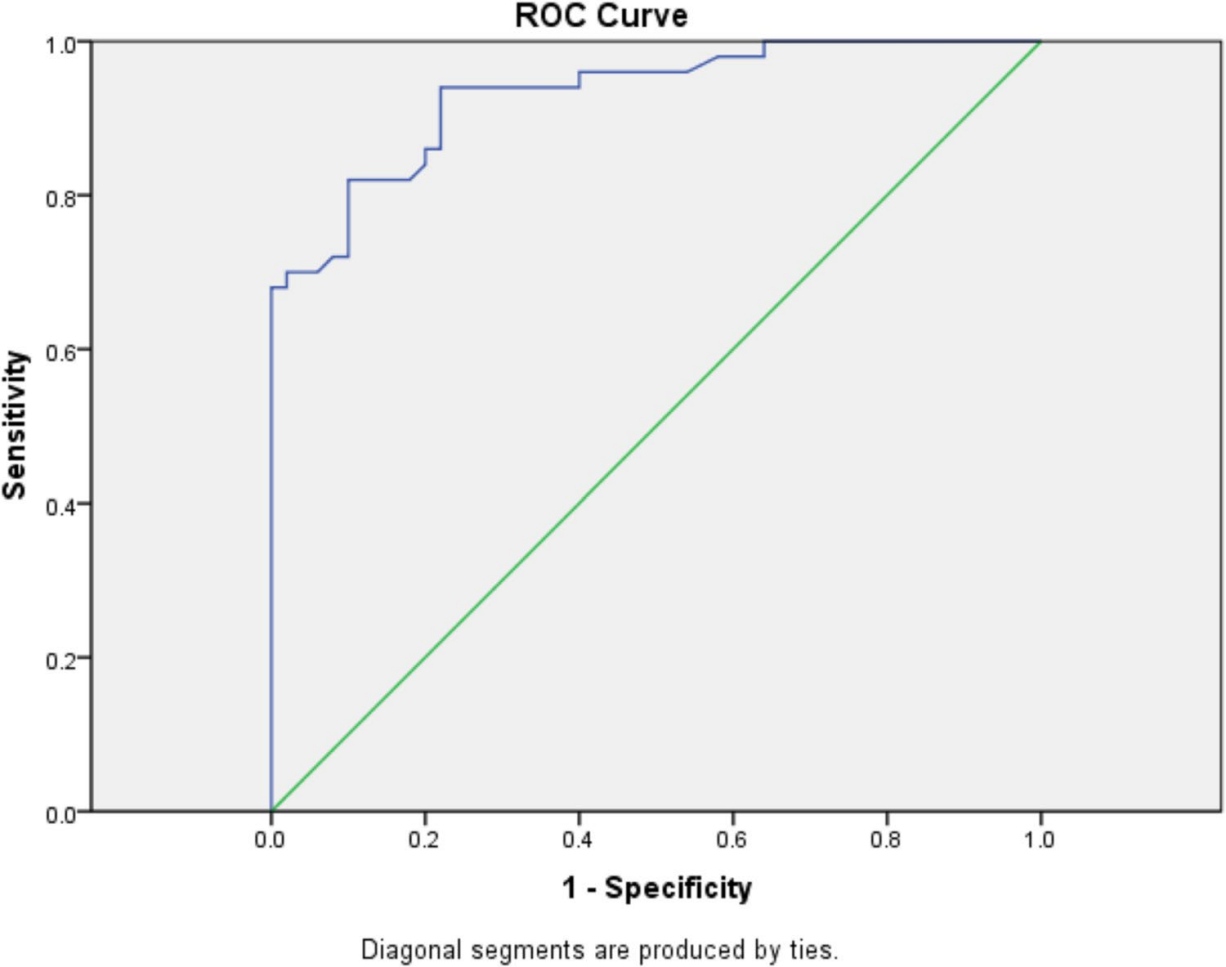
ROC curve to predict the diagnostic value of NEAT in psoriasis patients from controls. ROC curve showed that the expression of NEAT could be used as a predictor of psoriasis. NEAT had an area under the curve (AUC) of 0.931 (95% CI 0.884–0.978, p < 0.001)

 ROC analysis for lncRNA NEAT revealed that it could be considered as a good diagnostic marker for psoriasis. The best chosen cutoff level was 1.28, at which the AUC was 0.931, the specificity was 90% and the sensitivity was 82%. Figure 2; Table 4.

**Table 4:** ROC analysis of NEAT

AUC	P value	Cut off	Sensitivity	Specificity	95% Confidence interval	
					Lower bound	Upper bound
0.931	< 0.001	1.28	82%	90%	0.884	0.978

*AUC* Area under the curve

## Discussion

Psoriasis is an inflammatory, systemic disease characterized by an increase in the immune-related cells release of pro-inflammatory cytokines. Psoriasis not only dermatological disorder but also associated with many comorbidities, like psoriatic arthritis, cardiovascular and psychiatric complications [11].

In this study we found a state of dyslipidemia in psoriatic patients in the form of increased TG, cholesterol levels and decrease in HDL level. this is agreed with previous studies revealed elevated TG, cholesterol, LDL, and decreased HDL in psoriasis patients [12, 13] in addition many studies reported that dyslipidemia in psoriasis patients is associated with increase in the risk of cardiovascular disease [14, 15].

Interestingly, we found significant positive correlation between the dyslipidemia and the levels of inflammatory cytokine TNF and ROS; this may suggest that the dyslipidemia acts as initiating signal in chronic inflammatory process in psoriasis. These finding coincides with previous studies reported that the treatment of associated hyperlipidemia induces clinical improvement in psoriasis manifestation which could be attributed to anti-inflammatory and immunomodulatory effects [16]. Oxidative stress has been believed to be a key regulator in the pathogenesis of psoriasis. ROS produced by fibroblasts, keratinocytes, and endothelial cells produce chemotaxis on neutrophils and other inflammatory cells in addition to their accumulation in psoriatic lesions with subsequent excessive superoxide (O2−) production during phagocytosis [17].

We also found significant increase in TNF gene expression and ROS levels in psoriasis patients compared to the control, these are concordant with the previous studies reported that psoriasis is characterized by an abnormal differentiation and proliferation of keratinocytes, caused by a dysregulated auto-immune T cell response to several inflammatory cytokines [18]. TNFα, is secreted by both T cells and antigen-presenting cells within psoriasis skin lesion, more over TNF antagonists become a new modality in treatment of moderate-to-severe psoriasis [19]. TNF-α produces induction of adhesion molecules on vascular endothelial cells causing stimulation of keratinocytes production of other pro-inflammatory mediators [20]. Another study revealed significantly increased serum ROS like nitric oxide, malondialdehyde with decrease in the antioxidant activity of superoxide, catalase psoriasis patients compared to the control [21].

VEGF is a key growth factor that cause regulation of the neovascularization, during embryogenesis, and pathological processes [22]. We found significant up regulation of VEGF gene expression in psoriasis patients compared to controls, this is agreed with previous studies on psoriatic patients reported that serum levels of VEGF were significantly higher in psoriasis patients than control subjects and a highly significant correlation between VEGF and PASI score was found, suggesting that VEGF can be a good indicator for severity of the disease [23].

On an attempt to identify the epigenetic regulation of psoriasis pathogenesis, we investigated the expression of lncRNA NEAT and found significant up regulation of its expression in psoriasis patients compared to controls, NEAT is one of lncRNAs involved in inflammation and modulation of the immune system. NEAT are thought to maintain the integrity of the paraspeckles structure; up on inflammatory stimulation, NEAT is translocated to the cytoplasm, activating various caspases [24]. Moreover, previous studies revealed that NEAT has a role in cancers by regulating apoptosis and cell cycle progression in breast cancer cells [25].

Another study reported that NEAT has a critical role in regulation of cell growth, proliferation, apoptosis, invasion and metastasis. In addition, expression level of NEAT in tumor tissues associated with survival of cancer patients indicating that NEAT could be a therapeutic target in cancer treatment [26].

ROC curve was done to evaluate the diagnostic value of lncRNA NEAT as a predictor for discrimination between psoriasis patients and controls; At cut-off points of 1.28 at which the AUC was 0.931, the sensitivity was 82% and the specificity was 90%, NEAT could be considered a good diagnostic biomarker for psoriasis.

Apoptosis is a programmed cell death. dysregulated apoptosis can lead neoplastic or autoimmune diseases such as psoriasis. Caspase-3 is a marker of apoptosis [27]. we found significant increase in caspase 3 level in psoriasis patients compared to controls, this is in accordance with a study revealed that over expression of caspase-3 in the psoriatic lesion has a potential role in psoriasis pathogenesis and the positive correlation between the caspase-3 expression and poor prognostic of psoriasis lesion [28]. However contrary reports revealed that physiological apoptosis is crucial in normal epidermis development; and the induction of apoptosis is involved in the regression of psoriatic hyperplasia after PUVA therapy; moreover, decrease of physiological apoptosis in the psoriatic lesion could lead to psoriatic hyperplasia [29], this is also augmented by another report stated that apoptosis regulates keratinocyte proliferation. Dysfunctional apoptosis has a key role in hyper proliferation with incomplete differentiation of epidermal keratinocytes; further more psoriatic keratinocytes have the ability to resist apoptosis, which is a key regulator in development of psoriasis [30].

Thus, we can conclude that dyslipidemia is more prevalent in psoriasis patients. Dyslipidemia seems to act as initiator signals in development of psoriasis through secretion of inflammatory cytokines and increase the oxidative stress. Cytokines and ROS stimulate two pathways; the first one is modulation of immune cells and stimulation of keratinocyte proliferation and development of psoriatic lesion; the second pathway is the release of NEAT from paraspeckles to counter act the inflammation and activate apoptosis through induction of caspase-3. Thus, the increased expression of NEAT and caspase-3 levels seemed to be a protective and counteracting epigenetic regulator in psoriasis.

## Data Availability

The datasets generated during and/or analyzed during the current study are available from the corresponding author on reasonable request.
